# Pathogen screening of Zambian ticks: new insights on the occurrence of tick-borne pathogens in the country

**DOI:** 10.1186/s13071-026-07353-w

**Published:** 2026-04-10

**Authors:** Chikosenu Makayi, Simainga Simainga, Katja Mertens-Scholz, Lidia Chitimia-Dobler, Susanne Fischer, Marcelo B. Labruna, Ben J. Mans, George Dautu, Hanka Brangsch, Martin Simuunza, Martin H. Groschup, Ansgar Schulz

**Affiliations:** 1 Department of Disease Control, School of Veterinary Medicine, University of Zambia, Lusaka, Zambia; 2 Central Veterinary Research Institute, Lusaka, Zambia; 3 Institute of Bacterial Infections and Zoonoses, Friedrich-Loeffler-Institut, Jena, Germany; 4 Bundeswehr Institute of Microbiology, Munich, Germany; 5 Fraunhofer Institute of Immunology, Infection and Pandemic Research, Penzberg, Germany; 6 Experimental Parasitology, Department of Veterinary Sciences, Faculty of Veterinary Medicine, Ludwig-Maximilians-Universität, LMU, Munich, Germany; 7 Institute of Infectology, Friedrich-Loeffler-Institut, Greifswald, Insel Riems Germany; 8 Faculdade de Medicina Veterinária E Zootecnia, Universidade de São Paulo, São Paulo, Brazil; 9 Epidemiology, Parasites and Vectors, Agricultural Research Council-Onderstepoort Veterinary Research, Onderstepoort, South Africa; 10 Department of Life and Consumer Sciences, University of South Africa, Florida/Roodepoort, South Africa; 11 Department of Zoology and Entomology, University of the Free State, Bloemfontein, South Africa; 12 Institute of Novel and Emerging Infectious Diseases, Friedrich-Loeffler-Institut, Greifswald, Insel Riems Germany

**Keywords:** Zambia, Ticks, Wild life, Cattle, Flagging

## Abstract

**Background:**

Ticks are important ectoparasites for both humans and animals and can also transmit a wide range of different viral, bacterial, and parasitic pathogens, which are commonly known as “tick-borne pathogens” (TBPs). In Zambia (Southern Africa), a number of studies have been conducted on TBPs, but information on their distribution and genetic variation is still incomplete.

**Methods:**

Between 2022 and 2023, 588 ticks were collected in 3 provinces from the environment/vegetation via flagging as well as from cattle and wild host species (buffaloes and tortoises). After tick species identification and DNA/RNA extraction, the samples were tested for viral (orthonairo-, flavi- and arenaviruses) and bacterial (*Rickettsia*, *Anaplasma*, *Ehrlichia*, and *Coxiella*) pathogens using different polymerase chain reaction (PCR) assays. Subsequently, positive samples were sequenced and analyzed on the basis of different genes (*rrs* (16S rRNA); 23S-5S intergenic spacer region; *ompB*; *gltA*; *sca4*).

**Results:**

Apart from some *Hyalomma* and *Amblyomma* species, most of the ticks collected belonged to the genus *Rhipicephalus,* in which *Rhipicephalus appendiculatus* made up the largest proportions. No viral RNA was detected inside the ticks, but *Rickettsia* (*Ri. africae*, *Ri. aeschlimannii*, *Ri. sibirica, Ri. tamurae*-like agent), *Anaplasma* (*An. bovis*, *An. marginale*, *An. platys, An. phagocytophilum*-like agent), and *Ehrlichia* species (*Eh. ruminantium*, *Eh. chaffeensis*-like agent) were most frequently found.

**Conclusions:**

Many of the pathogens found had already been described in Zambia. Nevertheless, there were some unexpected findings. The detection of *Ri. sibirica* (*ompB* and *23S-5S* spacer region; in ticks from cattle) or *Eh. chaffeensis*-like amplicons (*rrs*; in a questing *Rh. appendiculatus* tick) may suggest that some pathogens or closely related species might be more widespread than previously assumed. In addition to these rarely described pathogens in Africa, further studies should be carried out on the detection of TBPs of viral origin.

**Graphical Abstract:**

**Figure:**
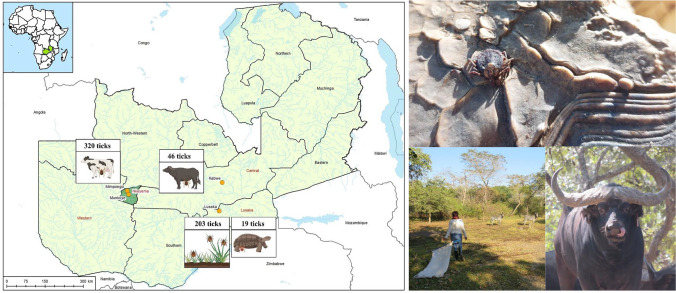

**Supplementary Information:**

The online version contains supplementary material available at 10.1186/s13071-026-07353-w.

## Background

Ticks are important ectoparasites for both humans and animals. They can also transmit a wide range of different viral, bacterial, and parasitic diseases, commonly known as tick-borne diseases (TBDs), while the causative agents are referred to as tick-borne pathogens (TBPs) [1]. In addition to the associated health risks following a tick bite, they also cause significant economic losses in livestock farming. This is due to the animals’ reduced fitness and blood loss during mass infestations. Although ticks are found almost worldwide, many regions still lack knowledge about tick species and their associated diseases [2]. One of those countries is Zambia in Southern Africa. Although there have been a number of studies on tick-borne pathogens in ticks and their hosts in the country, information on their distribution and genetic variation is still incomplete. Zambia’s tropical climate, diverse landscapes, intensive agriculture, and large number of wild animals in the national parks make it prone to the occurrence of ticks and their pathogens. The obligate intracellular Gram-negative *Rickettsia* species belonging to the Rickettsiaceae family are among the most important bacterial pathogens transmitted by ticks. Several *Rickettsia* species have been found in ixodid ticks and/or their hosts in Zambia, including cattle [3–6], dogs [4, 5, 7] and nonhuman primates [8]. They also have been detected in argasid ticks from warthog burrows, chicken coops, and bat guano [9, 10]. Although *Rickettsia* spp. has already been detected serologically in humans, no actual clinical case has been reported thus far [7, 11]. Other important causative agents of TBDs belong, for example, to the Anaplasmataceae and Coxiellaceae families (both obligate intracellular Gram-negative bacteria). *Anaplasma* has been found in ticks and their hosts in Zambia, including cattle [12, 13], dogs [14, 15], nonhuman primates [8], and antelopes [16]. *Anaplasma* was also detected in argasid ticks collected from chicken coops and warthog burrows [10], but no human cases of anaplasmosis have been reported in the country to date. The presence of *Ehrlichia* has been demonstrated in Zambian cattle [6, 12, 17], dogs [14], and in ticks from tortoises exported to Japan [18]. *Coxiella burnetii*, the causative agent of Q fever, was first detected in 2013 in cattle and goats [19]. The pathogen was later found in dogs and rodents [20]. Despite the first detection of *Co. burnetii* antibodies in humans back in 1999 [21], no actual human cases of Q fever have been reported in the country.

Certainly, the most relevant viral pathogen of zoonotic TBDs in sub-Saharan Africa is Crimean-Congo hemorrhagic fever virus (*Orthonairovirus haemorrhagiae*, CCHFV) [22]. *Hyalomma* ticks are considered the main vector and reservoir. While susceptible animals show no symptoms, humans can develop severe hemorrhagic fever syndromes with a high mortality rate during the course of the disease [23]. Although the virus is likely distributed across the entire African continent, it was only detected in ticks and Zambian cattle (serologically) in 2021 [24]. Since this report, neither human cases of CCHFV nor positive animals or ticks have been recorded in the country. Studies on other viral causative agents of TBDs have not been conducted at all [25].

This study aims to fill in the gaps regarding the spread and genetic diversity of TBD pathogens in Zambia. For this purpose, almost 600 ticks were collected and tested for various causative agents of TBDs. Half of the ticks were taken from cattle, similar to previous studies [3, 6, 16]. The rest were collected from buffaloes, tortoises, or directly from the environment via flagging. The flagged ticks were collected from private game ranches, which had a high abundance of native wildlife and no domestic livestock. In addition to the bacterial and viral pathogens mentioned, the ticks were also tested for other tick-borne orthonairoviruses. These included Nairobi sheep disease virus (*Orthonairovirus nairobiense*, NSDV) and Dugbe virus (*Orthonairovirus dugbeense*, DUGV). There are no classical representatives of TBDs belonging to the arenavirus and flavivirus families in sub-Saharan Africa. However, a subset of the samples was also screened for these pathogens.

## Methods

### Sampling sites and tick collection

**Fig. 1:**
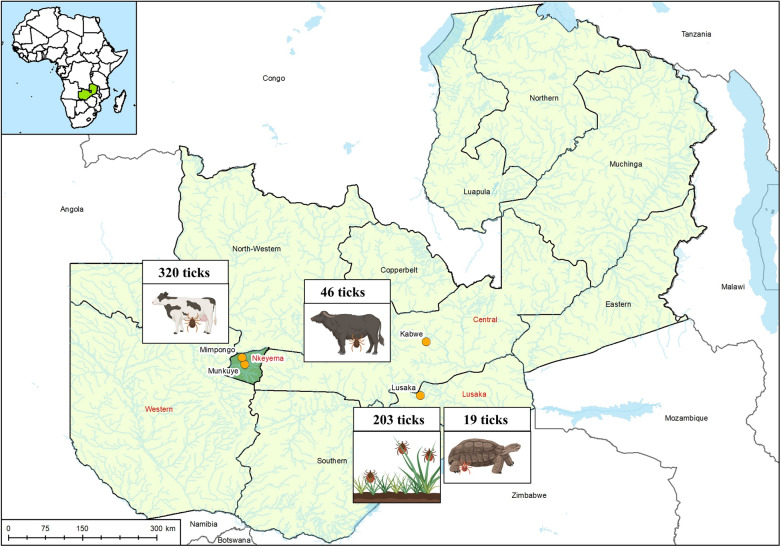
Map of Zambia showing the collection sites (Kabwe, Lusaka, Mimpongo, Munkuye) and sample types (ticks from cattle, buffaloes, tortoises, and vegetation). This map was created using ArcGIS^®^ software by Esri. ArcGIS^®^ and ArcMap™ are the intellectual property of Esri and are used herein under license. Copyright ^©^ Esri. All rights reserved. For more information about Esri® software, please visit www.esri.com.' The map was apdapted using BioRender.com

Between 2022 and 2023, a total of 588 ticks were collected from cattle in Nkeyema district (*n* = 320), African buffaloes near Kabwe (*n* = 46), leopard tortoises in a private zoo near Lusaka (*n* = 19), and from vegetation via flagging at two game lodges near Lusaka (*n* = 203; lodge A: 44, lodge B: 159) (Table 1). The 320 ticks collected from cattle (*Bos taurus*) in Nkeyema district were sampled using a targeted strategy, including only a subset of animals from farms near Munkuye and Mimpongo. Selection was based on the accessibility of the animals and the willingness of the owners to participate. Animals treated with acaricides within the past month to minimize bias in tick presence were excluded. Moreover, 46 ticks were collected from six female African buffaloes (*Syncerus caffer*) as part of a routine screening for foot-and-mouth disease (FMD) on a game and livestock farm near the town of Kabwe. In addition, a total of 19 ticks were collected from leopard tortoises (*Stigmochelys pardalis*) kept in a private zoo near the capital of Lusaka. The rest of the ticks (*n* = 203) were collected via flagging on private properties of two different game lodges near Lusaka (lodge A, *n* = 44 and lodge B, *n* = 159), which combine safari experiences with accommodation (Fig. 1). The grounds of lodge A were very wide with a variety of biotopes (savannah, bushland, and wetland) and were home to a whole range of different wild mammals (e.g., *Kobus vardonii*, *Connochaetes taurinus, Alcelaphus buselaphus lichtensteinii, Loxodonta africana, Giraffa* sp.), birds (e.g., *Grus carunculatus, Scopus umbretta*), and reptiles (e.g., *Varanus niloticus*). Remarkably, almost all adult ticks from lodge “A” were flagged at a single spot in a dense bushland around a few small trees, while almost no adult ticks were found at the rest of the lodge. On the contrary, lodge B was relatively small in comparison with lodge A and had more of the character of a large, fenced zoo enclosure. Only two large mammal species (*Aepyceros melampus*, *Equus quagga*) and semi-wild monkeys (*Chlorocebus pygerythrus*) were kept here.

**Table 1:** Overview of the different tick species collected from animals and vegetation by flagging

Tick species	Flagging		Collected from tortoises		Collected from buffaloes			Collected from cattle		Total	
	*n*	%	*n*	%	*n*		%	*n*	%	*n*	%
*Rhipicephalus appendiculatus*	197	97	0	0	7		15.2	62	19.4	266	45.2
*Rhipicephalus microplus*	0	0	0	0	0		0	162	50.6	162	27.6
*Rhipicephalus evertsi evertsi*	1	0.5	0	0	24		52.2	42	13.1	67	11.4
*Rhipicephalus decoloratus*	0	0	0	0	14		30.4	0	0	14	2.4
*Rhipicephalus sculptus*	1	0.5	0	0	1		2.2	0	0	2	0.3
*Rhipicephalus simus*	1	0.5	0	0	0		0	1	0.3	2	0.3
*Rhipicephalus* sp.	3	1.5	0	0	0		0	0	0	3	0.5
*Hyalomma truncatum*	0	0	0	0	0		0	27	8.4	27	4.6
*Hyalomma rufipes*	0	0	0	0	0		0	10	3.1	10	1.7
*Amblyomma nuttalli*	0	0	19	100	0		0	0	0	19	3.2
*Amblyomma variegatum*	0	0	0	0	0		0	16	5	16	2.7
Total	203		19			46		320		588

Afterward, the ticks were stored at −80 °C and put in 80% ethanol along with cold packs shortly before being shipped to Germany.

### Tick analysis

#### Tick identification, nucleic acid extraction, and blood-meal analysis

All ticks were morphologically identified using the identification keys of Apanaskevich and Horak [26], Apanaskevich and Horak [27], and Walker and Keirans [28]. Afterward, individual ticks were homogenized in AVL buffer (Qiagen, Hilden, Germany) using a TissueLyser II (Qiagen, Hilden, Germany) (300 µl AVL buffer + 1 × 5 mm steel bead). The homogenates were centrifuged (10 min/8,000 rpm) and 100 µl of supernatant was used for nucleic acid extraction. DNA/RNA was extracted using a KingFisher Flex device (ThermoFisher, Waltham, USA) with the NucleoMag® VET kit (Macherey–Nagel, Düren, Germany) according to the manufacturer’s protocol. To verify the morphological identification, a subset of ticks was analyzed by partial Sanger sequencing (Eurofins, Luxembourg, Luxembourg) of the mitochondrial 16S rRNA amplicon as described elsewhere [29], which was amplified using the SuperScript III PCR kit (ThermoFisher, Waltham, USA). In addition, the extracted RNA/DNA from flagged adult ticks was used for blood meal analyses on the last blood meal in the previous nymphal stage. PCR specific for mitochondrial cytochrome b from wildlife species [30] was performed using the QuantiTect Multiplex PCR NoROX Kit (QIAGEN, Hilden, Germany) as previously described. The PCR products were verified by gel electrophoresis in a 1.5% agarose gel and sequenced by Sanger sequencing (Eurofins, Luxembourg, Luxembourg).

#### Screening for viral pathogens

All ticks were screened for CCHFV [31], NSDV, and DUGV using RT-qPCR [32, 33]. Flaviviruses were detected using a Pan-Flavi melting curve PCR [34], while various orthonairoviruses [35] and arenaviruses [36] were screened using two different conventional PCR assays, as previously described. Amplicons of positive samples were subsequently sequenced by Sanger sequencing (Eurofins, Luxembourg, Luxembourg). Inactivated cell culture supernatants of the respective pathogens were used as positive controls, whereas water served as a negative control.

#### Screening for bacterial pathogens

All collected samples were screened by quantitative real-time PCR (qPCR) for *Rickettsia* spp. (*gltA*), *Anaplasma* spp. (*rrs* (16S rRNA)), *Anaplasma centrale* (*groEL*), *Anaplasma marginale* (*msp1b*), *Anaplasma platys* (*rrs*), *Anaplasma phagocytophilum* (*msp2*), and *Co. burnetii* (IS1111) [37–42].

For the species-specific *Anaplasma* qPCR assay, Cq values greater than 35 were considered questionable or likely negative result. For a phylogenetic analysis of *Anaplasma* and *Ehrlichia*, a conventional PCR targeting a fragment of the *rrs* gene was also performed [43], followed by Sanger sequencing. These sequences were used for the phylogenetic trees. Samples that tested positive but could not be clearly identified by PCR or sequencing were further analyzed using additional conventional PCR and nested PCR, targeting the *Anaplasma*/*Ehrlichia* spp. *rrs* [44] and *An. phagocytophilum groEL* genes [45, 46], respectively. All resulting PCR amplicons were also sequenced by Sanger sequencing.

For species differentiation of rickettsiae, the *rrs*, *gltA*, *sca4*, and *ompB* genes, as well as the 23S-5S intergenic spacer region, were amplified and sequenced [43, 47–49]. The *ompB* gene sequences were used to build a phylogenetic tree.

Synthetic DNA from the respective pathogens was used as a positive control for the qPCR assays, whereas verified positive samples from previous field studies were used as positive controls for conventional PCR assays. In both cases, water served as the negative control.

#### Phylogenetic analyses

The PCR product sequences were used as queries in an online Basic Local Alignment Search Tool (BLAST; National Center for Biotechnology Information, Bethesda, MD) search [50] via Geneious Prime 2021.0.1 (https://www.geneious.com). Accession numbers (acc. no.) of sequences that were uploaded to the NCBI GenBank (National Center for Biotechnology Information, Bethesda, MD) and included in the analysis can be found in Table S1.

The PCR product sequences and publicly available reference sequences including a suitable outgroup (acc. nos.: OQ908861; CP001079; M73221) were aligned by MAFFT v7 (online) [51]. The alignments were analyzed by maximum likelihood analysis using RAxML v8.2.12 [52] with the GTRGAMMA model of evolution, i.e., the general time reversible (GTR) model of nucleotide substitution with the Γ model of rate heterogeneity, and 1000 bootstrap replicates. The trees were visualized by FigTree v1.4.3 (https://github.com/rambaut/figtree).

## Results

### Tick identification and blood-meal analysis

Most of the ticks collected belonged to the genus *Rhipicephalus*, with *Rhipicephalus appendiculatus* (*n* = 266) being most abundant, followed by *Rhipicephalus microplus* (*n* = 162) and *Rhipicephalus evertsi evertsi* (*n* = 67) (Table 1). In both private game lodges, *Rh. appendiculatus* was collected almost exclusively via tick flagging. In comparison, the range of tick species collected from animals was considerably more heterogeneous. In addition to other *Rhipicephalus* species (including *Rh. evertsi evertsi* and *Rh. microplus*), *Hyalomma rufipes*, *Hyalomma truncatum*, and *Amblyomma variegatum* were also collected from cattle, whereas only ticks of the genus *Rhipicephalus* were collected from buffaloes. All ticks collected from tortoises belonged to the species *Amblyomma nuttalli*. No results were obtained from the blood meal analysis of the flagged ticks.

### Screening for viral pathogens

After screening all the ticks for the various viruses, including CCHFV, NSDV, DUGV, flaviviruses, arenaviruses, and other orthonairoviruses, no viral RNA was detected.

### Screening for bacterial pathogens

#### *Rickettsia* spp.

**Fig. 2:**
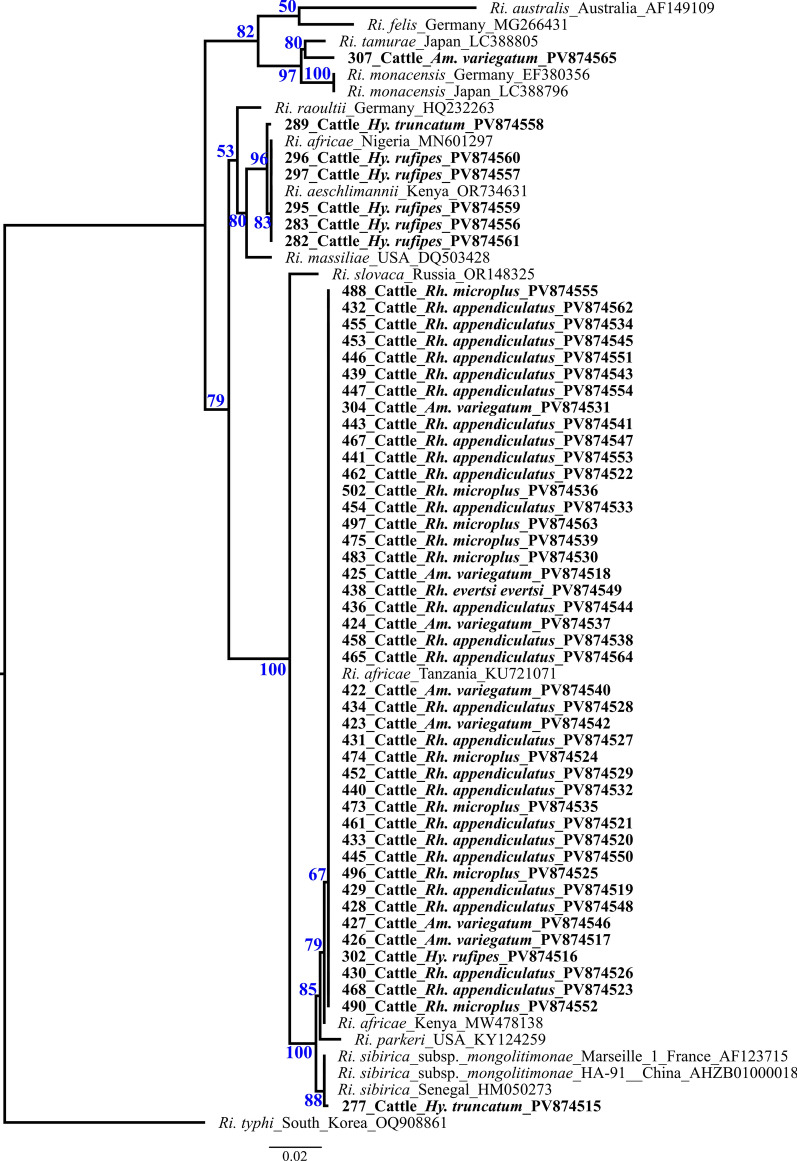
Maximum likelihood tree using RA×ML v8.2.12 on the basis of the alignment of *ompB* amplicons from ticks that tested positve for *Rickettsia* rooted to *Ri. typhi* (OQ908861). The bar indicates base substitutions per site. Blue numbers give branch support values. Field samples from this study are indicated in bold

A total of 65 ticks tested positive for *Rickettsia* spp. using a *Rickettsia* genus-specific qPCR (*gltA* gene) for screening. These ticks were exclusively collected from cattle in the Nkeyema district (Western Province). For species differentiation, conventional PCR assays targeting fragments of four rickettsial genes (*rrs*, *gltA*, *sca4*, *ompB*) were attempted, with DNA sequencing and BLAST analysis of PCR products. Among the 65 qPCR-positive ticks, successful amplification and reliable sequences were obtained for the *rrs* (56 samples), *gltA* (50 samples), *sca4* (19 samples), and *ompB* (64 samples) genes. Overall, it was possible to sequence at least one of the genes in 64 out of 65 samples (*ompB*). Only in one sample could the species not be unambiguously identified due to insufficient sequencing quality (Table 2). Results of BLAST analyses for the 64 tick samples are presented in Table S2. Most of the positive samples were identified as *Rickettsia africae* (56/65) and were found across all tick genera (*Rh. appendiculatus*, *Rh. evertsi evertsi*, *Rh. microplus*, *Hy. rufipes*, and *Am. variegatum*). *Rickettsia aeschlimannii* was the second most common species (6/65), but only in ticks of the genus *Hyalomma* (*Hy. rufipes* and *Hy. truncatum*), which were collected only from cattle. Furthermore, two other species of *Rickettsia* were found that were most closely related to *Rickettsia sibirica* (1/65; in *Hy. truncatum*) and a *Rickettsia tamurae* (1/65; in *Am. variegatum*). Due to the poor quality of the obtained 23S sequences, *Ri. sibirica* and *Ri. tamurae* can only be verified to a limited extent on the basis of the 23S-5S intergenic region (Table S2). Figure 2 shows the *ompB*-based phylogeny of *Rickettsia* spp., with bootstrap support values (1000 replicates) ranging from 50% to 100% shown at the nodes.

**Table 2:** Rickettsia-specific genes for species identification

*Rickettsia* species	Sequenced genes of *Rickettsia*-positive samples (*n* = 65)			
	16S	*gltA*	*sca4*	*ompB*
*Ri. africae*	55/65	50/65	18/65	56/65
*Ri. aeschlimannii*	1/65	0/65	1/65	6/65
*Ri. sibirica*	0/65	0/65	0/65	1/65
*Ri. tamurae*	0/65	0/65	0/65	1/65
*Rickettsia* sp*.*	0/65	0/65	0/65	0/65
Total	56/65	50/65	19/65	64/65

#### *Anaplasma *spp. and* Ehrlichia *spp.

A total of 38 samples tested positive by *rrs*-qPCR screening for the *Anaplasmataceae* family (includes genera *Anaplasma* and *Ehrlichia*) (Table 3). Of these, 34 originated from ticks that had been collected from cattle, tortoises, and buffaloes (*Bo. taurus*, *n* = 23; *St. pardalis*, *n* = 6; *Sy. caffer*, *n* = 5). The remaining four positive samples derived from ticks that were flagged at game lodge B. The four *Anaplasma*-specific qPCRs yielded positive results for some of the samples tested (Table 3). For *An. phagocytophilum*, only two samples were positive (albeit both with a Cq > 35). In total, 3 ticks tested positive for *An. centrale* (2 with a Cq > 35), 4 ticks for *An. marginale*, and 12 for *An. platys* (2 with a Cq > 35). The *rrs* (16S rRNA) sequences of the positive samples were analyzed and compared using BLAST (Table 3 and Table S2). On the basis of this analysis, 4 samples were identified as *An. marginale* (99.8–100%), 1 as *Anaplasma bovis* (99.7%), and 12 as *Ehrlichia ruminantium* (99.5–100%). Sample no. 212 showed the highest similarity to *An. phagocytophilum* (96.9%), and sample no. 169 the highest similarity to *Ehrlichia chaffeensis* (98.5%). Since sample no. 212 only shared 96.9% similarity with *An. phagocytophilum*, it was additionally tested using the second *rrs* (16S rRNA) PCR and the *groEL* nested PCR. While the *An. phagocytophilum*-specific *groEL* PCR was negative, the sequenced *rrs* (16S PCR) amplicon showed the highest similarity to *Anaplasma* sp. (98.11%) in the BLAST analysis (Table S2).

**Table 3:** Results of *Anaplasma* and *Ehrlichia* screening of the ticks

Positive samples 16S *Anaplasmataceae* qPCR		Cq values of specific qPCR					Sanger sequencing
Sample no./tick species	Host/collection type		*Anaplasma phagocytophilum*	*Anaplasma centrale*	*Anaplasma marginale*	*Anaplasma platys*	*rrs* (16S) (Pairwise identity)
60/*Rh. appendiculatus*	Flagging		–	–	–	–	–
132/*Rh. appendiculatus*	Flagging		–	–	–	–	–
169/*Rh. appendiculatus*	Flagging		–	–	–	–	*Eh. chaffeensis* (98.5%)
199/*Rh. appendiculatus*	Flagging		–	–	–	34.5	–
206/*Am. nuttalli*	*Stigmochelys pardalis*		–	–	–	34.9	–
211/*Am. nuttalli*	*Stigmochelys pardalis*		–	–	–	33.7	–
212/*Am. nuttalli*	*Stigmochelys pardalis*		–	–	–	29.2	*An. phagocytophilum* (96.9%)
219/*Am. nuttalli*	*Stigmochelys pardalis*		–	–	–	34.2	–
220/*Am. nuttalli*	*Stigmochelys pardalis*		–	–	–	(39.5)	–
221/*Am. nuttalli*	*Stigmochelys pardalis*		–	–	–	35	–
231/*Rh. evertsi evertsi*	*Syncerus caffer*		–	–	–	30.8	*An. bovis* (99.7%)
235/*Rh. evertsi evertsi*	*Syncerus caffer*		(38.1)	–	29.6	–	*An. marginale* (100%)
236/*Rh. appendiculatus*	*Syncerus caffer*		–	–	30.9	–	*An. marginale* (100%)
237/*Rh. appendiculatus*	*Syncerus caffer*		–	–	29.8	–	*An. marginale* (100%)
244/*Rh. sculptus*	*Syncerus caffer*		–	31.7	31.1	–	*An. marginale* (99.8%)
344/*Rh. appendiculatus*	*Bos taurus*		–	(35.7)	–	32.1	–
345/*Rh. appendiculatus*	*Bos taurus*		–	(38)	–	–	–
349/*Rh. appendiculatus*	*Bos taurus*		–	–	–	(39.5)	–
385/*Rh. microplus*	*Bos taurus*		–	–	–	–	–
395/*Rh. microplus*	*Bos taurus*		–	–	–	–	–
423/*Am. variegatum*	*Bos taurus*		–	–	–	–	*Eh. ruminantium* (100%)
424/*Am. variegatum*	*Bos taurus*		–	–	–	–	–
425/*Am. variegatum*	*Bos taurus*		–	–	–	–	*Eh. ruminantium* (100%)
426/*Am. variegatum*	*Bos taurus*		–	–	–	–	–
427/*Am. variegatum*	*Bos taurus*		–	–	–	–	–
432/*Rh. appendiculatus*	*Bos taurus*		–	–	–	–	*Eh. ruminantium* (100%)
433/*Rh. appendiculatus*	*Bos taurus*		–	–	–	–	–
434/*Rh. appendiculatus*	*Bos taurus*		–	–	–	(38.3)	*Eh. ruminantium* (100%)
438/*Rh. appendiculatus*	*Bos taurus*		–	–	–	–	*Eh. ruminantium* (100%)
440/*Rh. appendiculatus*	*Bos taurus*		(37.3)	–	–	–	*Eh. ruminantium* (100%)
441/*Rh. appendiculatus*	*Bos taurus*		–	–	–	–	*Eh. ruminantium* (100%)
448/*Rh. appendiculatus*	*Bos taurus*		–	–	–	–	*Eh. ruminantium* (100%)
455/*Rh. appendiculatus*	*Bos taurus*		–	–	–	–	*Eh. ruminantium* (100%)
462/*Rh. appendiculatus*	*Bos taurus*		–	–	–	–	*Eh. ruminantium* (100%)
468/*Rh. appendiculatus*	*Bos taurus*		–	–	–	(38.5)	–
469/*Rh. appendiculatus*	*Bos taurus*		–	–	–	–	*Eh. ruminantium* (100%)
476/*Rh. microplus*	*Bos taurus*		–	–	–	–	–
562/*Rh. microplus*	*Bos taurus*		–	–	–	–	*Eh. ruminantium* (99.5%)

Furthermore, some of the ticks appeared to be positive for more than one *Anaplasma* species (e.g., sample no. 231; no. 244). In one case (sample no. 212, from a tortoise), the *Anaplasma*-specific qPCR result did not match the Sanger sequencing result (*An. platys* versus *An. phagocytophilum*). While the *Eh. chaffeensis*-like agent (no. 169) was found in a single flagged *Rh. appendiculatus* tick, *Eh. ruminantium* was only detected in ticks (*Am. variegatum*; *Rh. appendiculatus*; *Rh. microplus*) collected from cattle. All *Anaplasma*-positive samples derived from ticks collected from buffaloes (*Rh. appendiculatus*, *Rh. evertsi evertsi*, *Rhipicephalus sculptus*) and tortoises (*Am. nuttalli*), respectively.

**Fig. 3:**
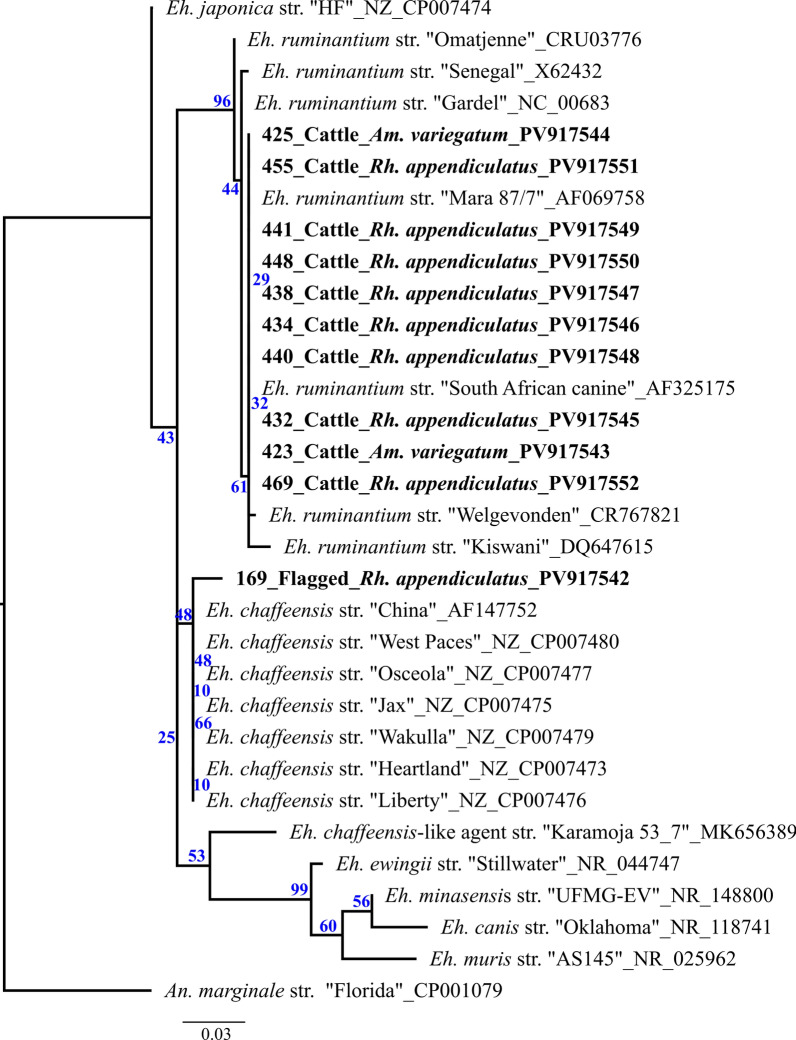
Maximum likelihood tree using RA×ML v8.2.12 on the basis of the alignment of 16S amplicons from ticks that tested positve for *Ehrlichia* rooted to *An. marginale* str. Florida (CP001079). The bar indicates base substitutions per site. Blue numbers give branch support values. Field samples from this study are indicated in bold

**Fig. 4:**
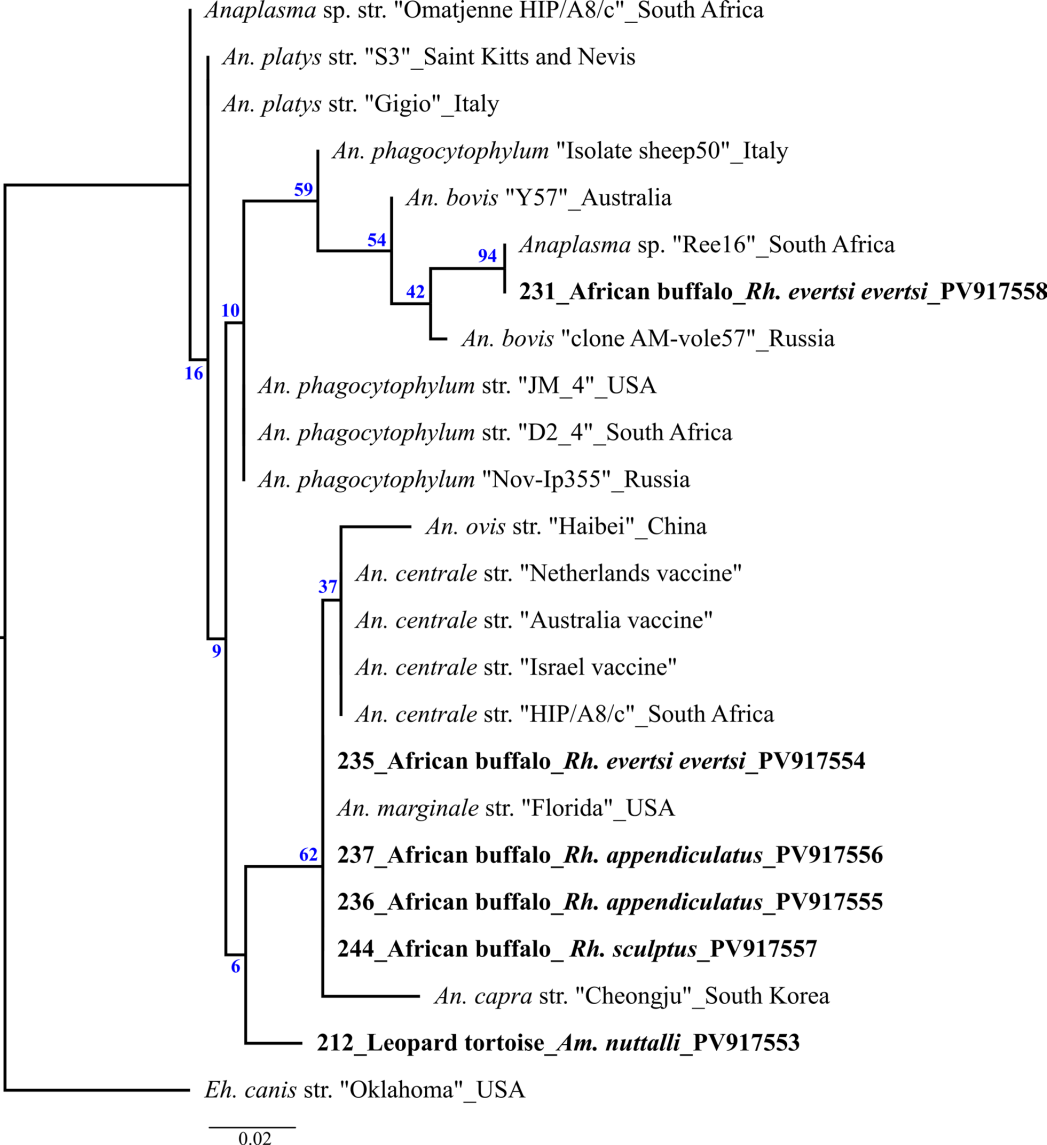
Maximum likelihood tree using RA×ML v8.2.12 on the basis of the alignment of 16S amplicons from ticks that tested positve for *Anaplasma* rooted to *Eh. canis* str. Oklahoma (M73221). The bar indicates base substitutions per site. Blue numbers give branch support values. Field samples from this study are indicated in bold

Figures 3 and 4 show the phylogenetic trees of *Anaplasma* and *Ehrlichia*, including all sequenced samples, with bootstrap support values (1000 replicates) widely ranging from 6% to 99%. Sample nos. 235–237 and 244 cluster unambiguously with the *An. marginale* reference sequence, while sample no. 231 aligns with an *An. bovis* sequence found in an oryx antelope from South Africa. Sample no. 212, on the contrary, clusters between *An. phagocytophilum* and *An. bovis* (Fig. 2). All *Eh. ruminantium*-positive samples clustered most closely with the two South African strains: Mara 87/7 and Pretoria North/South African Canine. Although sample no. 169 can be assigned to the *Eh. chaffeensis* group, it shows a greater divergence compared with the typical strain isolates (Fig. 3).

#### Coxiella burnetii

All ticks tested negative for *Co. burnetii.*

## Discussion

The examination of flagged ticks can provide more meaningful results than those obtained from blood-fed ticks collected from hosts. Through flagging, we collected mainly *Rh. appendiculatus* species (*n* = 197), a common tick species in southern/eastern Africa that mainly infests cattle, buffaloes, and antelopes [53]. Since many wild animals, including antelopes, lived at both collection sites, this result is not surprising. Pathogens were detected in two adult *Rh. appendiculatus* specimens from lodge B using qPCR (*An. platys*) and sequencing (*Eh. chaffeensis*-like agent), while all ticks collected at lodge A tested negative. *Anaplasma platys* is the causative agent of infectious canine cyclic thrombocytopenia (ICCT), with *Rhipicephalus sanguineus* sensu lato considered its main vector, but it has also been detected in other *Rhipicephalus* species [54]. It is widespread in the tropics and has already been detected in Zambian dogs [14, 15] and sable antelopes [16]. Unfortunately, sequencing was not possible, probably due to the high Cq value of 34.46, and the result should be interpreted with caution.

*Ehrlichia chaffeensis* causes human monocytotropic ehrlichiosis (HME) and has been described almost exclusively in the USA in the past. *Amblyomma americanum* is the only proven vector for this bacterium, whose major vertebrate host and reservoir is the white-tailed deer (*Odocoileus virginianus*) [55]. According to older data from the USA, HME has a hospitalization rate of 41–63% and a lethality rate up to 2.7% [56], making it a significant zoonotic pathogen. In the last decades, however, increasing numbers of suspected serological and antigenic findings have been recorded in other parts of the world [55]. On the African continent, the alleged presence of *Eh. chaffeensis*(-like) has only been detected in few countries (e.g., in ticks collected from ruminants in South Africa [57] or Uganda [58]). In Cameroon, an *Eh. chaffeensis*-like agent was also detected in humans with febrile symptoms [59] as well as in ticks collected from dogs [60]. Evidence that an *Eh. chaffeensis*-like pathogen might be present in Zambia has already been provided indirectly through the detection in ticks collected from tortoises that had been exported from Zambia as pets to Japan [18] and directly by detection in bovine blood samples from the Northern Province in Zambia [12]. However, these observations are based on small DNA sequence fragments of single or few genes, which is not sufficient for conclusive evidence of the presence of *Eh. chaffeensis* outside North America. Similarly to sequence MK656389 (*Eh. chaffeensis*-like agent str. Karamoja 53_7), which was obtained from ticks collected from cattle in Uganda, our sample also does not show 100% identity (Table 3 and S2) to the North American *Eh. chaffeensis* strains (Fig. 3). This could indicate that the pathogen (along with the other sequences found in Africa) may be closely related to *Eh. chaffeensis* and is not *Eh. chaffeensis* itself. It is still completely unknown which tick species or vertebrate hosts could be involved in the transmission pathway of such an agent in Africa. The detection of the agent in a flagged *Rh. appendiculatus* tick is particularly meaningful. It can be assumed that the tick was transstadially infected, making this species a potential candidate for the maintenance of an *Eh. schaffeensis*-like agent in its distribution area in southern/eastern Africa. However, as our data do not provide enough information to draw conclusions about vector competence, further epidemiological surveys and experimental infection studies are required to obtain a more accurate understanding of the actual dissemination of *Eh. chaffeensis*-related agents in Africa.

All ticks collected from leopard tortoises (*St. pardalis*) were identified as *Am. nuttalli*. This is a highly specialized tick species from southern Africa whose adult stages exclusively infest tortoises, with *St. pardalis* being one of the main hosts [61]. Data on the occurrence of pathogens in wild reptiles, including *St. pardalis* and their associated ticks in Africa, are very limited. For example, *Eh. ruminantium* was detected in *Amblyomma sparsum* collected from leopard tortoises that were shipped from Zambia to the USA in the late 1990 s [62]. In addition, various *Rickettsia* species, and as already mentioned, a sequence similar to *Eh. chaffeensis*, were detected in *Amblyomma* ticks collected from animals of the same species after being exported as pets to Japan between 2004 and 2009 [18]. Finally, a study from South Africa [63] has shown the presence of REP (“reptile group”) *Borrelia* and *Coxiella*-like endosymbionts in *Amblyomma* ticks also collected from *St. pardalis*, while another survey from Kenya [64] described the occurrence of *Eh. ruminantium*, *Hepatozoon fitzsimonsi*, and *Paracoccus* sp. In this study, we were able to detect different *Anaplasma* species in the *Am. nuttalli* ticks collected from leopard tortoises. One tick was even found to harbor an agent closely related to *An. phagocyophilum* (Table 3 and Fig. 4), the causative agent of human granulocytic anaplasmosis (HGA). However, since the Cq values were relatively high and the sequencing of sample no. 212 did not match the qPCR result (*An. platys* versus *An*. *phagocytophilum*), it is difficult to draw a definite conclusion. Moreover, the *An. phagocytophilum*-specific *groEL* PCR was negative, and the sequencing results for the two *rrs* (16S rRNA) PCR amplicons were ambiguous. Apart from the presence of *An. platys*, another pathogen similar to *An. phagocytophilum* may also have been present in the tick.

The African buffalo (*Sy. caffer*) is a natural carrier of important infectious animal diseases (e.g., FMD, anaplasmosis, theileriosis, trypanosomiasis, Rift Valley fever virus (RVFV), etc.) indigenous to Africa, which often occur without serious symptoms. Thus, it can transmit various dangerous diseases to cattle in wildlife–livestock interface areas [65]. Previous studies on infectious diseases in African buffaloes in Zambia have mainly focused on FMD [66, 67] and theileriosis [68]. Apart from several *Rh. appendiculatus* specimens, we collected *Rh. evertsi evertsi* and *Rh. decoloratus* (African blue tick) from buffaloes (Table 1). These are two very common Afrotropical tick species that are known to infest not only cattle, but also African buffalo, among others [69]. Furthermore, a single *Rh. sculptus* tick, a rather rare species native to southern and eastern Africa [28], was found. *Anaplasma marginale* was detected in four ticks collected from buffaloes. It is considered the main causative agent of bovine anaplasmosis in cattle and can lead to severe disease and death, especially in older individuals [70]. It is known that many *Rhipicephalus* species are involved in the transmission cycle of (bovine) anaplasmosis [54]. Although the available data on *Rh. sculptus* do not provide information on diseases associated with this species [53], we found both *An. marginale* and *An. centrale* in one specimen collected from the buffaloes. Previous studies have shown that the significantly less pathogenic *An. centrale* often occurs as a co-infection with *An. marginale* in African buffalo [71]. In one *Rh. evertsi evertsi* tick, both *An. platys* and *An. bovis* were detected simultaneously, which could also indicate a co-infection of the tick and/or the buffalo with the two *Anaplasma* species. Considering the sampling bias (blood-fed ticks, only female buffalo sampled, lack of seasonality), which is often unavoidable when sampling wildlife, it should be noted that the results must be interpreted with caution [71]. However, it is suspected that the African buffalo serves as a reservoir for anaplasmosis, developing few to no symptoms. Although it is not known how likely transmission from buffalo to cattle is and under what circumstances spillover occurs [70], these findings underscore the recommendation to minimize the contact between buffalo and cattle in endemic areas [65].

The majority of the ticks found on cattle belonged to the genus *Rhipicephalus*, with *Rh. microplus* representing by far the largest proportion. This tick species, originally native to Asia, has spread across most tropical and subtropical regions of the world in recent decades, largely as a result of the global livestock trade [72]. It is considered to be the most important tick species in cattle production in the (sub-) tropics, primarily due to increased acaricide resistance and its aggressive occurrence, which causes major financial losses worldwide through declining animal productivity and disease transmission [72]. In addition, two *Hyalomma* species (*Hy. truncatum* and *Hy. rufipes*) and *Am. variegatum* were also collected, all of which are known to infest cattle herds in large numbers in Africa and thus can lead to the similar problems [69]. In 12 ticks, the presence of *Eh. ruminatium* was demonstrated. This pathogen, which is frequently found in Africa, can cause heartwater disease (or cowdriosis) in domestic and wild ruminants and is mainly transmitted via *Amblyomma* ticks, with *Am. variegatum* and *Amblyomma hebraeum* being attributed a central role. Due to its high mortality rate, it can cause considerable economic damage to cattle stocks, especially in high-yielding breeds [73]. Phylogenetic analysis based on *rrs* amplicons revealed that all positive *Eh. ruminantium* samples clustered most closely with two South African strains, Mara 87/7 and Pretoria North/South African Canine. Of these, pathogenicity in cattle was described at least for the Mara strain. However, we are not aware of any clinical symptoms in the animals before or after sampling. Although a review by Makala and Mangani [74] published in 2003 concluded that heartwater disease is widespread in Zambia and poses a major economic problem, only a few studies [12] have been published in the meantime about the disease distribution and occurrence of different strains.

While previous studies [3–5, 8, 10, 11] have demonstrated the presence of various *Rickettsia* species (*Ri. africae*, *Rickettsia massiliae*, *Rickettsia lusitaniae*, *Rickettsia hoogstraalii*, *Ri. conorii* ssp. *caspia*, *Ri. aeschlimannii*-like species, *Rickettsia felis*, *Rickettsia asembonensis*) in Zambia, no reliable scientific data on human cases have been reported in the country [25]. Most recently [75], a report from Japan indicated that a traveler, following the appearance of characteristic clinical symptoms, tested positive after her return from Zambia for *Ri. conorii* ssp. *conorii*, one of the causative agents of Mediterranean spotted fever (MSF).

Of all the ticks examined in this study, only those collected from cattle were tested positive for *Rickettsia*. Most of these could be assigned to *Ri. africae* (56/65), the causative agent of African tick bite fever (ATBF). The two Afrotropical *Amblyomma* species *Am. variegatum* and *Am. hebraeum* are considered to be the main vectors and reservoirs. We have found the pathogen in various tick species including *Am. variegatum*, but since only blood-fed ticks were analyzed, it is not possible to draw any conclusions about vector competence. Given the typically mild course of the disease, ATBF is often undiagnosed in endemic countries. Therefore, it seems to be in line with the general pattern of this disease that despite this large number of positive ticks, no human cases of ATBF have been reported in the regions where the samples were taken [76].

Furthermore, *Ri. aeschlimannii* was detected in 6/65 ticks. The sequences of *ompB* amplicons showed a high similarity with isolates from Kenya. This pathogen is primarily associated with ticks of the genus *Hyalomma*. Interestingly, we found *Ri. aeschlimannii* only in *Hyalomma* ticks collected from cattle. However, since the ticks were collected directly from the host, we cannot determine whether the *Rickettsia* DNA originates from the cattle. *Rickettsia aeschlimannii* was mainly described in the Mediterranean region [77], but there are an increasing numbers of records of *Ri. aeschlimannii* detections in sub-Saharan Africa [78, 79], including Zambia [3, 6]. It is believed that *Ri. aeschlimannii* can also cause MSF-like disease with mild symptoms in humans when transmitted through the bite of an infected tick. On the basis of previous findings [3, 6], these results confirm that the pathogen is circulating in the country.

The detection of a *Ri. sibirica ompB* gene sequence in one of the ticks is particularly noteworthy. This pathogen was first described in France in 1996 [80] in a human patient. Since then, more and more cases have been reported in the Mediterranean region. It typically causes a mild, nonfatal disease, but some complications have been described, such as septic shock, disseminated intravascular coagulation, neurological disorders, acute renal failure, and myopericarditis [81]. Only very few cases have been reported in Africa, e.g., from Cameroon [82] or South Africa [83], which is why this finding from Zambia represents a valuable contribution to the sparse data on the occurrence of the pathogen in Africa.

*Rickettsia tamurae* actually originates from Japan and is considered pathogenic to humans [84]. Different haplotypes of *Ri. tamure*-like agents have been reported in ticks from Central [85] and South America [86]. There are no data on its occurrence in Africa. The discovery of a *Ri. tamurae*-like strain in Zambia is thus a special case. Since only the sequencing of the *ompB* gene was successful (the quality of the 23S sequences was limited), conclusions regarding *Ri. sibirica* and *Ri. tamurae*-like agent must be interpreted cautiously. Further research is needed on the distribution of these pathogens in sub-Saharan Africa to make more precise statements about their epidemiology.

The limitations of this study include, on the one hand, the lack of seasonal data, and on the other hand, the fact that samples were only collected from a geographically restricted area. The ticks were not collected year-round, thus any potential seasonal patterns could not be recorded. This might be one of the reasons why viral RNA was not detected in any of the ticks examined. In addition to the lack of seasonality, it is possible that the lower stability of RNA viruses compared with bacteria may have led to false-negative results, even though the cold chain was maintained throughout the sample collection. Since most of the ticks collected originate from host animals, and blood-fed ticks may contain host DNA/RNA, the data do not allow conclusions to be drawn regarding vector competence or potential reservoir species. Although detection in adult questing ticks can provide valuable data, as it can be assumed that at least one transstadial transmission has occurred, it cannot replace complex in vitro infection studies on vector competence. It must also be noted that the phylogeny of *Anaplasma* and *Ehrlichia* is based on the 16S amplicon (Figs. 3 and 4). Due to its conserved nature, it is suitable for an initial broad classification but not for in-depth phylogenetic analysis (as also reflected by the low bootstrap values) [87]. This is particularly important to consider when evaluating the findings of these two pathogens, which show apparent genetic similarity to *An. phagocytophilum* and *Eh. chaffeensis*, respectively. Overall, this highlights the efforts that will be required in Zambia and beyond in the future. Large-scale studies conducted over longer periods, focusing on collecting questing ticks and identifying the pathogens they carry, will be necessary. Even though this approach is significantly more resource-intensive than sampling host animals, it remains the only viable option for addressing the fragmented data on tick-borne pathogens (TBP) across the African continent.

## Conclusions

This study highlights the prevalence of tick-borne pathogens in Zambia, detecting several known pathogens, including Rickettsia (Ri. africae, Ri. aeschlimannii, Ri. sibirica, Ri. tamurae-like), Anaplasma (An. bovis, An. marginale, An. platys, An. phagocytophilum-like), and Ehrlichia (Eh. ruminantium, Eh. chaffeensis-like).Unexpected findings, such as Ri. sibirica in cattle ticks and an Eh. Chaffeensis -like agent in questing Rh. appendiculatus, suggest some pathogens may be more widespread than previously thought. Although no viral RNA was detected, further research is needed to explore the genetic variation, distribution, and potential new viral TBPs. Limitations include the lack of seasonal data and geographically restricted sampling, which could have affected pathogen detection. Additionally, reliance on blood-fed ticks limits conclusions about vector competence and reservoirs. To gain a clearer understanding of tick-borne pathogen dynamics, future large-scale, long-term studies with targeted viral screening are essential for Zambia and sub-Saharan Africa.

## Supplementary Information

Supplementary Material 1. Table 1

Supplementary Material 2. Table 2

## Data Availability

All data generated or analyzed during this study are included in this published article and its supplementary information files.
